# Earlier or heavier spinal loading is more likely to lead to recurrent lumbar disc herniation after percutaneous endoscopic lumbar discectomy

**DOI:** 10.1186/s13018-022-03242-x

**Published:** 2022-07-16

**Authors:** Fei Wang, Kai Chen, Qiushui Lin, Yuegang Ma, Hao Huang, Chuanfeng Wang, Ping Zhou

**Affiliations:** 1 Department of Orthopedic Surgery, Shaoxing People’s Hospital, Shaoxing, Zhejiang Province China; 2 Department of Orthopedics, Shanghai Changhai Hospital of Naval Medical University, Shanghai, China; 3 Department of Orthopedic Surgery, Tenth Affiliated Hospital of Tongji Medical University, Shanghai, China

**Keywords:** Percutaneous endoscopic discectomy, Lumbar disc herniation, Recurrence, Risk factor

## Abstract

**Purpose:**

To evaluate the clinical features of and risk factors for recurrent lumbar disc herniation (rLDH) after percutaneous endoscopic lumbar discectomy (PELD) in our clinical practice.

**Methods:**

A total of 942 consecutive patients who underwent single-level PELD from January 2013 to August 2019 were included. Patients were divided into the recurrence group and the nonrecurrence group. Patient characteristics, radiographic parameters and surgical variables were compared between the two groups. Univariate analysis and multiple logistic regression analysis were adopted to determine the risk factors for recurrence after PELD.

**Results:**

The prevalence of rLDH was 6.05%. Age, sex, tobacco use, duration of low back pain, body mass index (BMI), occupational lifting, herniated disc type, facet joint degeneration, operation time and time to ambulation were significantly different between the two groups. Univariate analysis showed that age (*P* < 0.001), sex (*P* = 0.019), BMI (*P* = 0.001), current smoking (*P* < 0.001), occupational lifting (*P* < 0.001), facet joint degeneration (*P* = 0.001), operation time (*P* = 0.002), and time to ambulation (*P* < 0.001) could be significantly associated with the incidence of rLDH after PELD. Multivariate analysis suggested that an older age (*P* < 0.001), the male sex (*P* = 0.017), a high BMI (*P* < 0.001), heavy work (*P* = 0.003), grade II facet joint degeneration (*P* < 0.001) and early ambulation (*P* < 0.001) were significantly related to rLDH after PELD.

**Conclusions:**

An older age, the male sex, a higher BMI, heavy work, grade II facet joint degeneration, and early ambulation are independent significant risk factors for rLDH after PELD. Great importance should be attached to these risk factors to prevent rLDH. We suggest that patients control their weight, avoid heavy work, ambulate at an appropriate time, and perform strengthening rehabilitation exercises to reduce the incidence of rLDH.

## Introduction

Lumbar disc herniation (LDH) is one of the most common spinal degenerative diseases, presenting as symptoms of persistent lower back pain and/or sciatica. More than 90% of patients with LDH improve with conservative treatment [1]. Spine surgeons often recommend surgical treatment for patients with LDH when standard conservative treatment fails. Compared with conservative treatment, surgical management is a more effective method for alleviating pain and improving physical functions [2]. Lumbar discectomy is the “gold standard” for the treatment of LDH. With the improvement of less-invasive surgical techniques, open lumbar discectomy is no longer the only choice for the treatment of LDH.

Percutaneous endoscopic lumbar discectomy (PELD), as a minimally invasive technique, has been widely used in the treatment of LDH in our country. PELD yields clinical outcomes similar to those of microdiscectomy/open discectomy [2]. Meanwhile, PELD has the potential advantages of less blood loss, faster recovery of physical function, a shorter hospital stay, quicker pain relief and fewer complications [3, 4]. With the popularity of the PELD procedure, an increasing number of surgical complications have been reported, such as postoperative dysesthesia, nerve root injury, dural tears and recurrence [5].

Recurrent lumbar disc herniation (rLDH) after PELD is one of the most concerning complications for spine surgeons and is the most important factor affecting the surgical outcome. The literature indicates that the incidence of rLDH ranges between 0 and 11% [6–8]. Due to dural adhesion and scar formation, reoperation carries greater potential risks. Meanwhile, secondary surgery causes additional physical trauma for patients and leads to greater financial burdens on families and society. Thus, it is very important to explore risk factors for rLDH to prevent recurrence. Numerous studies have found that rLDH is caused by a variety of risk factors, including age, obesity, smoking, the experience of the surgeon, and the location of herniation [7–9]. However, previous studies have not been able to reach a consistent conclusion. Based on previous studies and our clinical experience, we further evaluated the risk factors for rLDH after PELD.

## Materials and methods

This was a retrospective study of consecutive patients over 18 years who underwent single-level PELD at two institutes (Shanghai Changhai Hospital of Naval Medical University and Tenth Affiliated Hospital of Tongji Medical University) from January 2013 to August 2019. The inclusion criteria were as follows: 1) lower back pain with radiation to the leg in a distribution corresponding to root compression on magnetic resonance imaging (MRI); 2) failure of 6 months of conservative treatment; 3) relief of leg pain immediately after PELD; 4) postoperative MRI showing complete removal of the herniated disc; 5) recurrence of the same symptoms as before the first operation; 6) disc herniation at the same level confirmed on MRI; and 7) 1-year follow-up data. The exclusion criteria included surgical site infection, central stenosis, lumbar instability, spondylolisthesis, acute trauma, history of previous spinal surgery and spinal metastasis in the involved segments. The study was approved by the clinical research ethics committee of our hospitals.

### Clinical and radiographic assessment

We investigated the patients’ demographic parameters, such as age, sex, and body mass index (BMI); comorbidities, such as diabetes mellitus; smoking and drinking status; occupational lifting; symptoms, such as the preoperative duration of low back pain, preoperative duration of leg pain, and preoperative visual analogue scale (VAS) score; surgical procedural details, such as the operation time, operative segment, and surgical approach; and preoperative radiographic parameters, such as the herniation type, degree of disc degeneration, grade of facet degeneration, facet orientation (FO), and facet tropism (FT).

The degree of disc degeneration was assessed on T2-weighted sagittal sequences according to the modified Pfirrmann criteria [10]. The grade of facet joint degeneration was evaluated on axial T2-weighted MRI according to the system described by Weishaupt et al. [11]: grade 0, normal; grade 1, mild degenerative disease; grade 2, moderate degenerative disease; and grade 3, severe degenerative disease. The facet joint angle (FO and FT) was measured on axial computed tomography images using bone windows and the method described by Li et al. [12].

All parameters were evaluated by two independent researchers. An experienced surgeon calibrated the parameters when two researchers disagreed with the data.

### Statistical analysis

Statistical analysis was performed utilizing SPSS statistical software 19.0 (Chicago, IL, USA). Clinical and radiographic parameters were compared by unpaired t test and Mann–Whitney U test when appropriate. Categorical variables were analysed by Chi-squared test. Univariate and multiple logistic regression analyses were adopted to identify risk factors for rLDH. *P* < 0.05 was considered statistically significant.

## Results

**Fig. 1:**
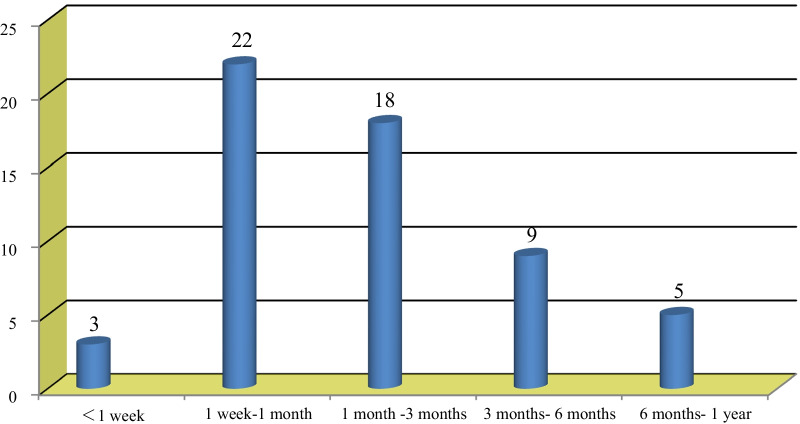
Distribution of recurrent patients followed up within one year after PELD

A total of 942 patients who were surgically treated at two institutes for LDH were identified retrospectively. A total of 837 patients were treated with percutaneous transforaminal endoscopic discectomy (PTED), while 105 patients were treated with percutaneous interlaminar endoscopic discectomy (PIED). There were 57 patients in the recurrence group, including 39 males and 18 females, with a mean age of 56.7 ± 8.7 years. The number of patients with recurrence in the first week, the first week to the first month, the first month to the third month, the third month to the sixth month, and the sixth month to the first year after minimally invasive surgery was 3, 22, 18, 9 and 5, respectively. There were 885 patients in the nonrecurrence group, including 462 males and 423 females, with a mean age of 41.2 ± 11.4 years. There was a significant difference in age, sex, tobacco use, duration of low back pain, BMI, occupational lifting, herniated disc type, facet joint degeneration, operation time and time to ambulation (the beginning of a long period of upright activity, aside from necessary eating or defecating in the early stage), between the two groups (Fig. 1; Table 1).

**Table 1:** Risk factors for recurrent lumbar disc herniation after PELD using univariate analysis

Variable	Recurrent group (*N* = 57)	Nonrecurrent group (*N* = 885)	*P* value
Age (year)	56.7 ± 8.7	41.2 ± 11.4	**< 0.001**
*Gender*			
Male	39(68.4)	462(52.2)	**0.017**
Female	18(31.6)	423(47.8)	
BMI (kg/cm^2^)	25.3 ± 2.9	23.6 ± 3.5	**< 0.001**
Tobacco use	33	261	**< 0.001**
Alcohol use	9	120	0.635
Diabetes mellitus	6	110	0.836
Duration of low back Pain (month)	29.4 ± 13.2	25.2 ± 17.5	**0.026**
Duration of leg pain (month)	16.8 ± 7.4	15.0 ± 7.3	0.081
Preoperative VAS for back	4.9 ± 1.2	5.0 ± 1.0	0.681
Preoperative VAS for leg	6.6 ± 1.2	6.5 ± 1.0	0.462
Sedentary occupation	18	201	0.125
Occupational lifting	21	111	**< 0.001**
*Herniated disc location*			
Central	6(10.5%)	69(7.8%)	0.256
Paramedian	42(73.7%)	732(82.7%)	
Foraminal	6(10.5%)	45(5.1%)	
Extreme lateral	3(5.3%)	39(4.4%)	
*Herniated disc type*			
Protrusion	11(19.3%)	225(25.4%)	**0.011**
Extrusion	33(57.9%)	432(48.8%)	
Sequestration	13(22.8%)	228(25.8%)	
*Pfirrmann Grading System*			
Grade I	1(1.8)	27(3.1)	0.101
Grade II	12(21.1)	237(26.8)	
Grade III	12(21.1)	294(33.2)	
Grade IV	20(35.1)	219(24.7)	
Grade V	8(14.0)	78(8.8)	
Grade VI	4(7.0)	30(3.4)	
Modic change	12	138	0.275
*Facet joints degeneration*			
Grade 0	5(8.8)	165(18.6)	< 0.001
Grade I	15(26.3)	318(35.9)	
Grade II	29(50.9)	223(25.2)	
Grade III	8(14.0)	179(20.2)	
FO (°)	46.3 ± 5.7	47.4 ± 5.2	0.160
FT (°)	3.92 ± 2.18	4.42 ± 2.13	0.091
*Operative segment*			
L2-L3	0	3	0.550
L3-L4	0	18	
L4-L5	30	405	
L5-S1	27	459	
*Surgical approach*			
PTED	51	786	0.878
PIED	6	99	
Operation time (minute)	82.4 ± 21.2	87.4 ± 10.5	**0.001**
Time to ambulation (day)	17.0 ± 7.2	24.3 ± 4.3	** < 0.001**

Bold symbols indicated there was statistically significance

*BMI* Body mass index, *VAS* Visual analogue scale, *FO* Facet orientation, *FT* Facet tropism, *PTED* Percutaneous transforaminal endoscopic discectomy, *PIED* Percutaneous interlaminar endoscopic discectomy

The results of the univariate logistic regression analysis of relevant clinical parameters (age, sex, BMI, tobacco use, duration of low back pain, occupational lifting, herniated disc type, facet joint degeneration, operation time, and time to ambulation) are summarized in Table 2. The results showed that age (*P* < 0.001), sex (*P* = 0.019), BMI (*P* = 0.001), current smoking (*P* < 0.001), occupational lifting (*P* < 0.001), facet joint degeneration (*P* = 0.001), operation time (*P* = 0.002), and time to ambulation (*P* < 0.001) were significantly associated with the incidence of rLDH. The herniated disc type was not found to be a risk factor for predicting recurrence after PELD (Table 2).

**Table 2:** Risk factors for recurrent lumbar disc herniation after PELD using univariate logistic regression analysis

Variable	OR	95%CI	*P* value
Age	1.115	1.087–1.144	**< 0.001**
gender	0.504	0.284–0.895	**0.019**
BMI	1.145	1.059–1.237	**0.001**
Smoking	0.304	0.176–0.525	**< 0.001**
Occupational lifting	0.246	0.139–0.436	**< 0.001**
Duration of low back pain	1.012	0.999–1.026	0.077
Herniated disc type	1.065	0.731–1.552	0.744
Facet joints degeneration	1.424	1.086–1.869	**0.011**
Operation time	0.968	0.949–0.988	**0.002**
Time to ambulation	0.739	0.690–0.792	** < 0.001**

Bold symbols indicated there was statistically significance

*BMI* Body mass index, *OR* Odds ratio, *CI* Confidential index

In the univariate analysis, age, sex, BMI, current smoking, occupational lifting, facet joint degeneration, operation time, and time to ambulation were potential risk factors for recurrence after PELD. Therefore, these potential risk factors were analysed using the multivariate Cox regression model (Table 3). Multiple logistic regression analysis showed that an older age (*P* < 0.001), the male sex (*P* = 0.017), a high BMI (*P* < 0.001), heavy work (*P* = 0.003), grade II facet joint degeneration (*P* < 0.001) and early ambulation (*P* < 0.001) were significant risk factors for rLDH (Table 3).

**Table 3:** Risk factors for recurrent lumbar disc herniation after PELD using multiple logistic regression analysis

Variable	OR	95% CI	*p*
Age	1.678	1.447–1.946	**< 0.001**
Gender	0.169	0.039–0.732	**0.017**
BMI	1.726	1.377–2.162	**< 0.001**
Smoking	0.402	0.129–1.253	0.116
Occupational lifting	0.129	0.033–0.507	**0.003**
Facet joints degeneration	0.007	0.001–0.034	**< 0.001**
Operation time	0.995	0.963–1.028	0.744
Time to ambulation	0.722	0.646–0.807	**< 0.001**

Bold symbols indicated there was statistically significance

*BMI* Body mass index, *OR* Odds ratio, *CI* Confidential index

## Discussion

PELD is a typical minimally invasive discectomy procedure that avoids the destruction of posterior spinal tissue and can be a feasible alternative to the conventional posterior approach for LDH. China has made the most contributions, based on the number of publications, to the field of fully endoscopic spine surgery [13]. Meanwhile, increasing attention has been given to the surgical complications of PELD. rLDH is a common complication of PELD and the most common reason for an unsatisfactory outcome following PELD for LDH. However, there exists debate on the risk factors for rLDH after PELD, and it is very difficult to define these risk factors due to the involvement of various complicated factors. In our study, we found that 57 (6.05%) of 942 patients who underwent PELD experienced rLDH. Chan Hong Park et al. [8] suggested that the recurrence rate of LDH after PTED was 11%. However, Du et al. [6] did not observe the recurrence of LDH after PELD in their study. Therefore, there is no consensus on the recurrence of LDH after PELD. However, the rate of rLDH after PELD may not have any guiding significance for patients with LDH. Meanwhile, we further found that age, sex, BMI, occupational lifting, facet joint degeneration, and time to ambulation were significantly correlated with the incidence of rLDH.

Most studies have reported age as one of the most important risk factors for recurrence after PELD [8, 9]. Yao et al. [9] found that an age over 50 years was closely associated with rLDH after successful PELD. In our study, we found that the average age of the 57 patients with rLDH was 56.7 ± 8.7 years. According to a retrospective cohort study, patients who were 57 years or older showed a higher reoperation rate after PELD than those younger than 57 years [14]. Recurrence after PELD is the main factor of reoperation [14]. In general, the older the patient is, the higher the probability of recurrence; therefore, PELD may not be an appropriate option for elderly people.

Generally, spinal tissue degeneration becomes increasingly severe with age. Marinelli et al. [15] reported that the stage of disc degeneration was associated with ageing. Meanwhile, patients with rLDH have been reported to have significantly more severe disc degeneration before surgery than patients with no rLDH [16]. However, we did not find a significant difference in the classification of intervertebral lumbar disc degeneration between the recurrence and nonrecurrence groups; lumbar disc degeneration was not associated with recurrence after PELD. Intervertebral lumbar disc degeneration is commonly accompanied by Modic changes [17], and severe intervertebral lumbar disc degeneration is significantly more common in patients with Modic changes [18]. Additionally, Modic changes are significantly correlated with an increased rate of rLDH [19]. However, our study found no difference in Modic changes between the recurrence and nonrecurrence groups. This further shows that rLDH may be the result of multiple factors. Lumbar disc degeneration may not be an independent risk factor for recurrence after PELD.

In our study, there was a significant difference in facet joint degeneration between the recurrence and nonrecurrence groups, indicating that facet joint degeneration is a significant risk factor for recurrence after PELD. A study of the anatomy of the lumbar facet joints demonstrated a significant role in shear load–carrying capacity [20]. Meanwhile, it was concluded that the posterior elements of the lumbar spine were more efficient in resisting anterior and posterior shear loads [20]. Therefore, we considered that degeneration of the posterior rather than anterior elements has a greater impact on spinal stability. Spinal instability has been found to be related to the biomechanical stress on the affected disc, which may be related to rLDH [12]. In the present study, we found that rLDH after PELD was more likely in those with grade II facet joint degeneration. This is the first time that an association between facet joint degeneration and rLDH after PELD has been proposed. However, there were no differences in facet joint parameters (FO and FT) between the two groups. Therefore, further biomechanical studies of facet joints in rLDH need to be performed.

The BMI is a statistical index determined using a person's weight and height to provide an estimate of body fat in males and females of any age. Kim et al. [21] reported higher rates of recurrence after successful PELD among patients with higher BMIs. Yao et al. [9] suggested that BMI ≥ 25 was the most robust risk factor responsible for recurrence after PELD. A meta-analysis also showed that the prevalence of rLDH in obese patients (BMI ≥ 25) was significantly higher than that in patients with a normal BMI [22]. However, some studies have failed to demonstrate such a correlation between obesity and recurrence following lumbar microdiscectomy [23]. In our study, we found that patients with rLDH had higher BMIs than those without rLDH (25.3 ± 2.9 vs. 23.6 ± 3.5, *P* < 0.001). BMI is one of the most important risk factors for postoperative rLDH. Our results are consistent with those reported by Yao et al. [9], who considered that obesity was the most important risk factor for recurrence after PELD. While we certainly agree, we also believe that strict bed rest is very important to prevent postoperative recurrence. Meanwhile, we considered various explanations for the differences in the research results, as follows. First, rLDH is an adverse outcome caused by multiple factors, and different risk factors have different effects on recurrence. Second, individual differences among patients could lead to different results from various studies. Third, there are many kinds of minimally invasive discectomy procedures, such as PELD, microendoscopic discectomy (MED), and microdiscectomy, and each surgeon has a different learning history and experience level. These two factors play important roles in recurrence. Meanwhile, we found a significant correlation between occupational lifting and rLDH. Li et al. [12] also reported that heavy work increased the risk of recurrence after discectomy, and clinical research reported by Kong et al. [24] demonstrated that a high BMI and heavy physical load intensity increased the possibility of rLDH. Furthermore, Sohrab et al. [25] suggested that the force required for LDH in the human spine was inversely correlated with the degree of disc degeneration. However, if the disc is subjected to higher pressures, it will become more degenerated and therefore more likely to herniate through an annular defect. Therefore, we consider that the overloading of damaged intervertebral discs might lead to rLDH. Above all, weight loss is very important in the perioperative period for patients with LDH, and long-term heavy work should be avoided after lumbar discectomy.

There are no clear standards for the duration of bedrest after PELD. Some scholars have considered that early ambulation should be carried out after lumbar discectomy, as it is conducive to promoting the recovery of bodily functions [2, 3]. However, Kim et al. [7] suggested that because early ambulation would increase the load on the injured intervertebral disc and easily lead to rLDH, the time of ambulation should be delayed. Qin et al. [26] also reported that the time to first ambulation was an important factor affecting recurrence after PELD. The recurrence rate among patients who ambulated within the first day after the operation was significantly higher than that among other patients [26]. According to the characteristics of scarring and wound healing of fibrous soft tissue, we suggest that patients should stay in bed for at least 3 weeks. However, many patients often fail to do this. Therefore, we strictly require patients to stay in bed within the first day after PELD. Aside from eating or defecating, patients should rest in bed as much as possible. When patients get out of bed, they must wear lumbar support to reduce the load on the intervertebral disc and limit the duration of upright activities as much as possible. At the same time, patients should perform functional exercises of the back muscles and strength training of the limbs in bed. Although the outcomes of our study are consistent with those reported by Qin et al. [26], there are some differences between the two studies. First, there are differences in the number of cases, follow-up time and surgeon experience level between their study and our study. In particular, the number of cases in their study was small. Second, the definition of the time to ambulation after PELD is different; they defined the time to ambulation as the time until the first walk after the operation [26], while we defined the time to ambulation as the beginning of a long period of upright activity. In our study, we found that the average time to ambulation in patients with rLDH was 17 days, while the average time to ambulation in patients without recurrence was 24 days. The time to ambulation was closely associated with rLDH after successful PELD. Therefore, we suggest that later ambulation is helpful to reduce the rate of rLDH after PELD.

## Limitations

Our study has several potential limitations that should be pointed out. First, this was a retrospective, nonrandomized, controlled study and was subject to bias. Second, our study had a small sample size and short follow-up time. Due to the short follow-up time, there may have been some undocumented cases of recurrence after PELD. Third, rLDH after PELD is caused by many factors, and the list of parameters we included may not have been comprehensive. Nonetheless, a larger multicentre clinical study with a longer follow-up period is needed to further validate our clinical findings.

## Conclusions

The current study shows that an old age, the male sex, a higher BMI, heavy work, grade II facet joint degeneration, and early ambulation are independent significant risk factors for rLDH. Patients should attach great importance to these risk factors to prevent rLDH. The mechanism between these risk factors and rLDH requires further investigation. At the same time, we believe that external factors or incorrect postoperative rehabilitation exercise may play a key role in postoperative recurrence. We suggest that patients should control their weight, avoid heavy work and perform strengthening rehabilitation exercises to reduce the incidence of rLDH.

## Data Availability

The data that support the findings of this study are available from Shanghai Changhai Hospital, Shaoxing People’s Hospital and Tenth Affiliated Hospital of Tongji Medical University, but restrictions apply to the availability of these data, which were used under licence for the current study, and so are not publicly available. Data are however available from the authors upon reasonable request and with permission of the above three hospitals, China.

## Abbreviations

LDH: Lumbar disc herniation
PELD: Percutaneous endoscopic lumbar discectomy
rLDH: Recurrent lumbar disc herniation
BMI: Body mass index
FO: Facet orientation
FT: Facet tropism
PTED: Percutaneous transforaminal endoscopic discectomy
PIED: Percutaneous interlaminar endoscopic discectomy
